# Sol–Gel-Processed Y_2_O_3_ Multilevel Resistive Random-Access Memory Cells for Neural Networks

**DOI:** 10.3390/nano13172432

**Published:** 2023-08-27

**Authors:** Taehun Lee, Hae-In Kim, Yoonjin Cho, Sangwoo Lee, Won-Yong Lee, Jin-Hyuk Bae, In-Man Kang, Kwangeun Kim, Sin-Hyung Lee, Jaewon Jang

**Affiliations:** 1 School of Electronic and Electrical Engineering, Kyungpook National University, Daegu 41566, Republic of Korea; lth0313@knu.ac.kr (T.L.); khi5735@knu.ac.kr (H.-I.K.); chongo815wls@knu.ac.kr (Y.C.); sangw98@knu.ac.kr (S.L.); yongsz@knu.ac.kr (W.-Y.L.); jhbae@ee.knu.ac.kr (J.-H.B.); imkang@ee.knu.ac.kr (I.-M.K.); 2 The Institute of Electronic Technology, Kyungpook National University, Daegu 41566, Republic of Korea; 3 School of Electronics and Information Engineering, Korea Aerospace University, Goyang 10540, Republic of Korea; kke@kau.ac.kr

**Keywords:** sol–gel, Y_2_O_3_, RRAM, multilevel cell

## Abstract

Yttrium oxide (Y_2_O_3_) resistive random-access memory (RRAM) devices were fabricated using the sol–gel process on indium tin oxide/glass substrates. These devices exhibited conventional bipolar RRAM characteristics without requiring a high-voltage forming process. The effect of current compliance on the Y_2_O_3_ RRAM devices was investigated, and the results revealed that the resistance values gradually decreased with increasing set current compliance values. By regulating these values, the formation of pure Ag conductive filament could be restricted. The dominant oxygen ion diffusion and migration within Y_2_O_3_ leads to the formation of oxygen vacancies and Ag metal-mixed conductive filaments between the two electrodes. The filament composition changes from pure Ag metal to Ag metal mixed with oxygen vacancies, which is crucial for realizing multilevel cell (MLC) switching. Consequently, intermediate resistance values were obtained, which were suitable for MLC switching. The fabricated Y_2_O_3_ RRAM devices could function as a MLC with a capacity of two bits in one cell, utilizing three low-resistance states and one common high-resistance state. The potential of the Y_2_O_3_ RRAM devices for neural networks was further explored through numerical simulations. Hardware neural networks based on the Y_2_O_3_ RRAM devices demonstrated effective digit image classification with a high accuracy rate of approximately 88%, comparable to the ideal software-based classification (~92%). This indicates that the proposed RRAM can be utilized as a memory component in practical neuromorphic systems.

## 1. Introduction

Nonvolatile resistive random-access memory (RRAM) is a crucial element for the next generation of memory technologies. With its simple sandwich structure, RRAM offers high-speed performance, low power consumption, and exceptional scalability. These advantages help overcome the von Neumann bottleneck and pave the way for neuromorphic computing systems, which aim to mimic the data-processing capabilities of the human brain [1,2,3]. To accommodate large amounts of data within a limited substrate area, the density of memory devices must be considerably increased. Various approaches, such as complex 3D structures such as vertical RRAM or crossbar configurations, have been proposed to achieve higher storage density [4]. However, these methods often entail complex and costly fabrication processes. Alternatively, a simpler approach is to enable MLC operation with a single memory device. Furthermore, in the context of neuromorphic systems that rely on parallel computation, it is essential to emulate biological synapses, which possess analog weight values. In terms of integration density, a nonvolatile memory component with multilevel memory capabilities is best suited to simulate synapses in offline-based neuromorphic systems compared with the performance of conventional digital memory devices. To achieve multilevel cell (MLC) operation, first, a large high-resistance state (HRS)/low-resistance state (LRS) ratio is required for generating more intermediate levels and enhancing the stability of each level during MCL switching operation, allowing for high bit density. In addition, uniform SET/RESET operation and stable endurance characteristics are required. Many new strategies are being developed to enhance resistive switching properties, uniformity, and stability [5,6,7].

Numerous metal oxide materials, including ZrO_2_, HfO_2_, TiO_2_, and Y_2_O_3_, have been utilized as active channel layers in RRAM devices [8,9,10,11,12,13,14,15,16,17,18]. Among them, Y_2_O_3_ stands out due to its high dielectric constant and large optical band gap, making it a promising high-k candidate for replacing SiO_2_ in complementary metal-oxide-semiconductor processes in the industry. The fast ion movement within Y_2_O_3_ enables rapid RRAM operation. For integrated RRAM arrays, the “sneak path” issue must be addressed. One solution is to combine Y_2_O_3_ with a transistor, acting as a selector. Y_2_O_3_ layers can also serve as a passivation layer for transistors, ensuring stable operation while simultaneously functioning as an active channel layer for RRAM devices. This approach reduces the number of fabrication steps and enhances cost efficiency [19,20]. Various conventional vacuum-based deposition methods have been used to deposit metal oxide active channel layers. However, these methods are time-consuming and not cost-efficient. In contrast, the sol–gel process is a well-known technique for depositing metal oxide layers using solution-phase precursor solutions. These precursor solutions can be used as ink for printing or spin-coating, allowing for a large-area application at a lower cost [21,22].

Herein, sol–gel-processed Y_2_O_3_ films were employed as active channel layers for RRAM devices. The fabricated Y_2_O_3_ RRAM devices exhibited conventional bipolar memory operation without requiring a forming process, thanks to the presence of oxygen vacancies with top Ag electrodes. These devices showed a sharp transition from a HRS to a LRS or vice versa. To enable multicell operation, the set current compliance was varied. As the current compliance values increased, the resistance values obtained gradually decreased, resulting in intermediate values within the LRS and HRS suitable for multilevel cell (MLC) switching. In numerical simulations, the Y_2_O_3_ RRAM device with multilevel memory states effectively functioned as an artificial synaptic cell with long-term plasticity in offline-learning-based neural networks. The constructed neuromorphic system successfully recognized complex handwritten digit images with an accuracy of approximately 88%. This level of accuracy is comparable to that of an ideal software system.

## 2. Materials and Methods

Y_2_O_3_ RRAM devices were fabricated using a vertically stacked structure comprising glass, indium tin oxide (ITO) bottom electrodes, Y_2_O_3_, and Ag top electrodes (TEs). The fabrication process involved several steps:

- Cleaning the ITO-coated glass substrate: The substrate was immersed in acetone and sonicated for 10 min, followed by additional cleaning with deionized water. A UV/O_3_ treatment was then performed for 1 h to remove any remaining organic impurities.
- Y_2_O_3_ precursor solution preparation: A Y_2_O_3_ precursor solution (0.3 M) was created by dissolving yttrium (III) nitrate tetrahydrate (Y(NO_3_)_3_4H_2_O) in 2-methoxymethanol. The solution was sonicated for an additional 10 min to achieve a clear solution.
- Coating Y_2_O_3_ on the ITO/glass substrate: A tiny Kapton tape was used to define the contact window for the ITO bottom electrodes. The Y_2_O_3_ precursor solution was then spin-coated onto the cleaned ITO/glass substrate at 3000 rpm for 50 s. The Kapton tape was removed before the heating process.
- Thermal annealing: To evaporate the solvent, the coated substrate was annealed on a hot plate at 100 °C for 10 min. The coated substrate was thermally annealed in a furnace at 500 °C for 3 h in the presence of air to convert the Y_2_O_3_ precursor into Y_2_O_3_.
- Deposition of Ag TE: A shadow metal mask was placed in contact with the Y_2_O_3_ film to define the size of the electrode (30 μm × 30 μm). A 100-nm-thick layer of Ag was deposited onto the Y_2_O_3_ films using thermal evaporation at a rate of 1.8 Å/s under 5 × 10^−6^ torr.

After fabrication, the crystal structure properties of the Y_2_O_3_ film were investigated using grazing incidence X-ray diffraction (GIXRD, X’pert Pro, Malvern PANalytical, Malvern, UK; incident angle = 0.3°, Cu-Kα, λ = 1.54 Å). The optical characteristics of the sol–gel processed films were estimated using an UV–visible technique (UV–vis; LAMBDA 265, Waltham, MA, USA). Moreover, a field-emission scanning electron microscope (FE-SEM, Hitachi 8230, Hitachi, Tokyo, Japan) was used to estimate the thickness and surface roughness of the film. The chemical composition analysis was conducted using X-ray photoelectron spectroscopy (XPS; Nexsa, ThermoFisher, Waltham, MA, USA). The electrical characteristics, including the I–V curve, retention, and endurance, were measured at room temperature using a probe station (MST T-4000A, Hwaseong, Korea) and parameter analyzer (Keithley 2636B, Keithley Instruments, Cleveland, OH, USA).

## 3. Results and Discussion

**Figure 1:**
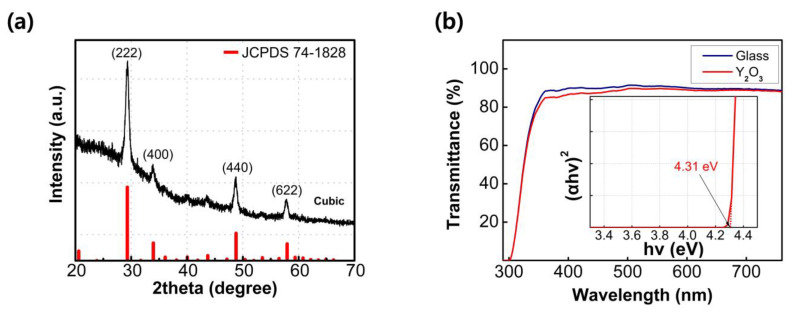
(**a**) GIXRD spectra of sol–gel-processed Y_2_O_3_ films and (**b**) transmittance spectra of sol–gel-processed Y_2_O_3_ films; the inset shows (ahv)^2^ vs. photon energy.

Figure 1a displays the GIXRD spectra of the Y_2_O_3_ film fabricated through the sol–gel method. The diffraction peaks at 2θ angles of 29.30°, 33.96°, 48.79°, and 57.93° correspond to the (222), (400), (440), and (622) crystallographic planes of Y_2_O_3_, respectively. Based on the GIXRD results, it is evident that the fabricated Y_2_O_3_ film consists entirely of a cubic crystal structure in the polycrystalline phase, specifically conforming to the cubic Y_2_O_3_ phase (JCPDS 74-1828). Notably, while a metastable monoclinic Y_2_O_3_ phase can form at low temperatures, the dominant phase observed here is the stable cubic Y_2_O_3_ phase [23]. The prominent peak at 29.30° indicates that the grains in the film primarily grow in the (222) plane. The crystallite size of the grains can be determined using the Scherrer equation.

D = (0.9 λ)/(β cos θ)(1)

where D, λ, β, and θ represent the crystalline size, CuΚα wavelength, full width at half maximum (FWHM) of the diffraction peak, and Bragg angle, respectively. The grain size of the (222) plane is the largest among the crystallographic planes, as evident from the narrowest FWHM of the diffraction peak at 29.30°. The calculated crystalline size from the (222) plane is 12.23 nm.

The optical characteristics of the Y_2_O_3_ film were investigated via ultraviolet–visible spectroscopy (UV–Vis). As shown in Figure 1b, the fabricated film shows a similar transmittance spectrum to that of the cleaned glass sample in the visible range, implying sufficient transparency of the fabricated film. The bandgap of the Y_2_O_3_ film can be obtained by extrapolating the linear part of the (ahv)^2^ versus hv graph. The inset of Figure 1b shows (ahv)^2^ versus hv derived from the Tauc equation.

(ahv)^2^ = A(hv − E_g_), (2)

where α denotes the absorption coefficient, A is a constant, h is the Planck constant, v is the photon frequency, and E_g_ is the optical bandgap. The intersection point between the extrapolation line and the *x*-axis represents the band gap of the Y_2_O_3_ film (a large optical band gap of 4.31 eV is observed in this case).

**Figure 2:**
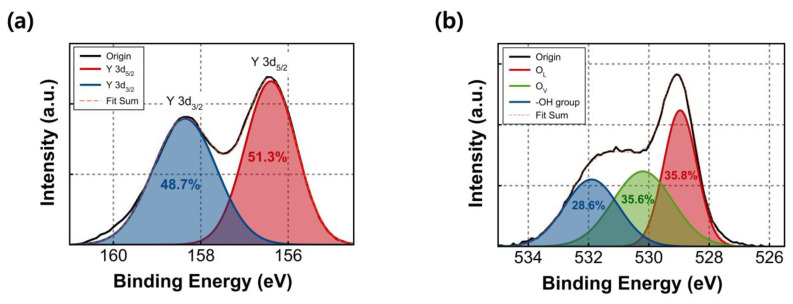
XPS spectra: (**a**) Y 3d and (**b**) O 1s of sol–gel-processed Y_2_O_3_ films.

Figure 2 presents the XPS results of the fabricated Y_2_O_3_ film, with all binding energy values corrected to the main C 1s peak (284.8 eV). In Figure 2a, the XPS spectra for Y 3d reveal the two splitting orbitals, Y 3d_5/2_ and Y 3d_3/2_. The Y 3d_5/2_ and Y 3d_3/2_ peaks are located at 156.4 eV and 158.4 eV, respectively, confirming the successful formation of the Y_2_O_3_ film on the substrate. The XPS results of Y_2_O_3_ film for O 1s are shown in Figure 2b. The deconvoluted O 1s peaks appear at 529.0, 530.2, and 531.9 eV, corresponding to the oxygen lattice (O_L_), oxygen vacancies (O_V_), and hydroxyl group (-OH), respectively. The area ratio of the O_L_ peak was 35.8%, while that of the O_V_ peak was 35.6%. These two components are almost equally proportioned in the O 1s spectra.

**Figure 3:**
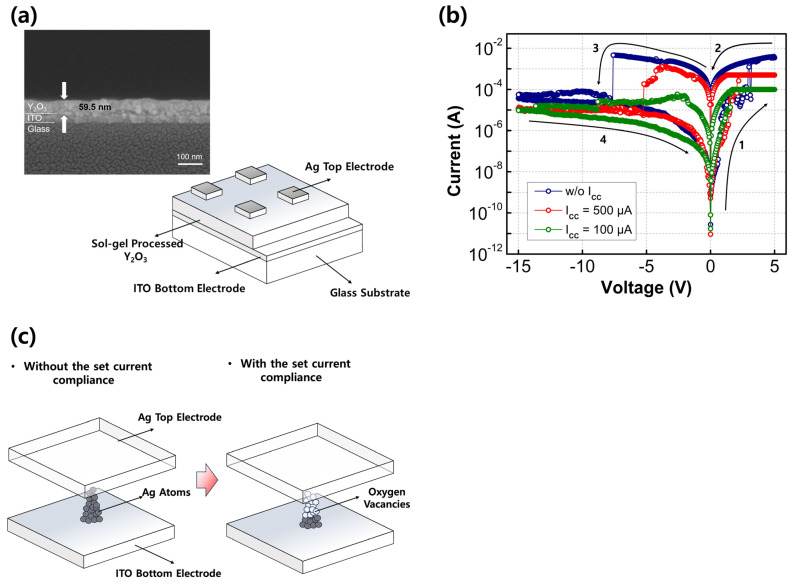
(**a**) Schematic of the device; the inset shows the cross-sectional SEM image of Y_2_O_3_ films on ITO/glass substrates; and (**b**) representative IV curves of the fabricated Y_2_O_3_ RRAM as a function of the set current compliance values. The arrows and numbers indicate the voltage sweep directions. (**c**) Schematic of the CF component change without/with the set current compliance.

Figure 3a presents a schematic of the fabricated RRAM devices; the inset shows the cross-sectional SEM image of Y_2_O_3_ films on ITO/glass substrates. Figure 3b displays representative I–V characteristics of the fabricated Y_2_O_3_ RRAM devices with different levels of compliance current. To observe the set/reset behavior, the voltage of the TE was swept from −15 V to 5 V, with the bottom electrode (BE) grounded. The read voltage was set at 0.1 V. The initially obtained resistance values were relatively high, indicating HRS. At a specific voltage known as the SET voltage, the current abruptly increased, resulting in low-resistance values, referred to as the LRS. When a voltage was applied from positive to negative values, known as the RESET voltage, the LRS turned back into the HRS. Notably, all fabricated Y_2_O_3_-based RRAM devices demonstrated apparent bipolar switching behavior without requiring an unwanted forming process. This distinguishes them from O_V_-rich metal-oxide-based RRAMs or Ag/Cu electrode-based RRAMs, often necessitating an additional forming process [24,25]. The Y_2_O_3_ films formed in this study exhibited some oxygen vacancy formation, and the use of Ag TE facilitated the easy formation of conductive filaments (CFs) without the need for a high-voltage forming process. This mechanism is based on conductive bridge random-access memory, which operates through the reduction and oxidation of Ag. Comparisons were made between Y_2_O_3_ RRAM devices with Ag TE and those with Au TE. The RRAM with Au TE did not exhibit evident RRAM characteristics since Au cannot form conductive bridges similar to Ag due to its low reactivity [14]. In contrast, the diffusive nature of Ag allowed it to penetrate the switching layer easily, enabling the formation of CFs. In addition, the Y_2_O_3_ switching layer contained abundant OV, which facilitated the formation of CFs without the need for applying a forming voltage. During the set process, a negative bias of −15 V was applied to the Ag TE, resulting in the HRS state. Upon applying a positive bias, Ag atoms lost electrons through an oxidation reaction and formed cations (Ag → Ag^+^ + e^−^). These cations then migrated to the BE and received electrons through a reduction reaction (Ag^+^ + e^−^ → Ag), leading to reduced Ag atoms in the switching layer. Once a particular voltage was reached, the TE and the BE were connected by conductive bridges made of Ag, transitioning the device to LRS. An abrupt increase in current at the SET voltage corresponds to the set process. Conversely, when the voltage applied to the TE was swept toward −15 V, the current dropped suddenly at the RESET voltage. This is attributed to the oxidation reaction of the Ag atoms, facilitated by a thermal electrochemical process. The oxidized Ag cations then returned to the TE, causing the conductive bridges to dissolve, thereby disconnecting the TE and the BE and returning the device to its initial HRS state.

When the fabricated RRAM device is operated without the set current compliance, the device experiences abrupt SET and RESET behaviors during positive and negative sweep cycles, respectively. With decreasing set current compliance values, the device experiences smooth SET and RESET behaviors during positive and negative sweep cycles, respectively. In our previous study, we confirmed that RRAM devices with an Ag/Y_2_O_3_/ITO structure are conductive bridge random-access memory devices, which rely on the migration of metal ions. Simultaneously, oxygen vacancy sites can also serve as pathways for filament formation because Ag ions can migrate through these sites with lower migration barriers, requiring less energy for movement. This behavior, affected by the set current compliance values, originates from the control of Ag filament generation. Without any set current compliance, the formed conductive filament is dominated by Ag. With decreasing set current compliance values, a portion of Ag is reduced, and O_V_ dominates the conductive filament. Owing to the restriction of Ag filament, the set current compliance values can be regulated. The dominant oxygen ion diffusion and migration within Y_2_O_3_ leads to the formation of O_V_ and Ag metal-mixed CFs between two electrodes. The filament composition changes from pure Ag metal to Ag metal mixed with O_V_, which is critical for realizing MLC switching by controlling the set current compliance values.

**Figure 4:**
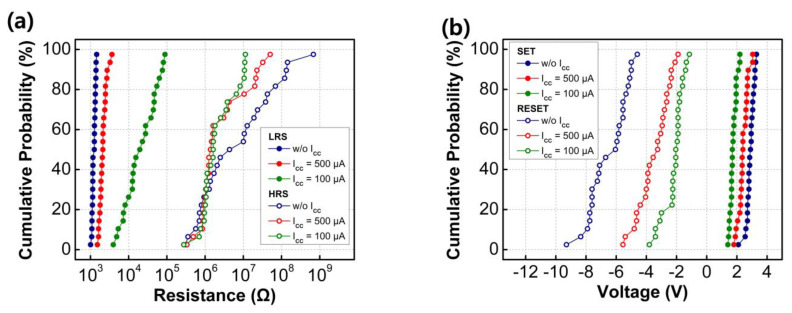
Cumulative distribution of resistance states (**a**) and SET and RESET values (**b**) for sol–gel-processed Y_2_O_3_ RRAM devices.

Figure 4a illustrates the cumulative distribution function of the resistance states of the fabricated RRAM devices, programmed by setting different current compliance levels with a read voltage of +0.1 V. With decreasing current compliance values, the fabricated RRAM devices showed the gradual resistance switching characteristics, indicating the formation of O_V_ and Ag metal-mixed CFs between two electrodes. This led to the formation of intermediate resistance values within the LRS and HRS, rendering them suitable for MLC switching. The LRS values are influenced by factors such as the radius of the CFs or the concentration of filaments between the bottom and TEs [26,27]. When a low compliance current is applied to the device, the formation of CFs is suppressed, leading to narrower CFs and a slight increase in the resistance of the switching layer. This phenomenon cannot occur when the conductive filament contains pure Ag filament. Conversely, the HRS values did not exhibit considerable changes, irrespective of the set current compliance value. The HRS values are known to be affected by the distance between the top of the broken CF and the TEs, known as the rupture region distance. The fact that the fabricated Y_2_O_3_ RRAM devices did not show considerable HRS variation indicates that the rupture region distance between the top of the broken CF and the TEs was unaffected by the set current compliance values.

With increasing set current compliance values, wider CFs are formed. Breaking thicker CFs, resulting from the reduction process, requires larger RESET voltages. Consequently, as the set compliance current values increase, the RESET voltage also increases [28]. By controlling the current compliance values, the fabricated RRAM devices can operate as a MLC with a capacity of 2 bits in one cell through three LRSs and one common HRS.

**Figure 5:**
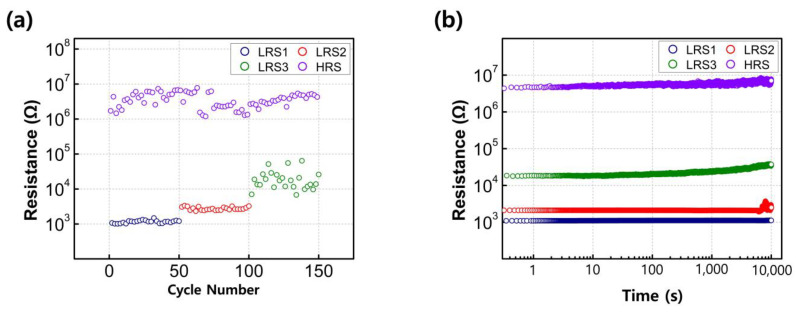

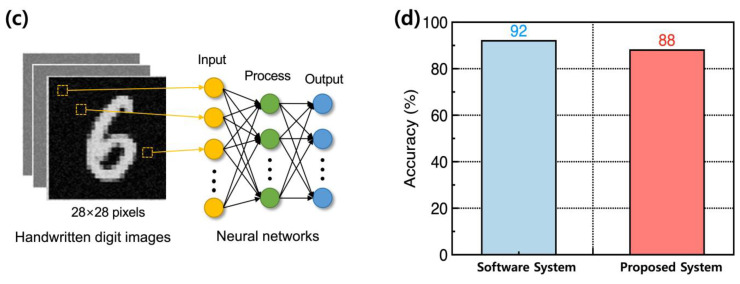
Nonvolatile properties of sol–gel-processed Y_2_O_3_ RRAM devices as a function of the set current compliance values: (**a**) endurance and (**b**) retention. (**c**) A constructed hardware neural network for classifying the handwritten digit images provided by the Modified National Institute of Standards and Technology. The system is composed of three different neuron layers corresponding to the input (784 neurons), processing (32 neurons), and output (10 neurons) signals. The neuron layers are interconnected through synaptic cells based on Y_2_O_3_ RRAM devices. (**d**) Recognition accuracy values for the ideal software system and the proposed hardware neural network. Each system was tested after training for 60 epochs.

To assess the reliability of the fabricated RRAM, endurance and retention tests were conducted under different compliance current conditions. The HRS and LRS values were extracted at a voltage of +0.1 V after programming and erasing operations, each lasting for 50 ms. As shown in Figure 5a, the resistance states of the fabricated RRAM devices, both with and without compliance current conditions, exhibited stable endurance properties for about 50 cycles. The largest on/off ratio was observed under compliance-current-free conditions, with a value of around 10^3^. The read-out margin, array size, and intermediate multilevels were decided by a HRS/LRS ratio [29,30,31]. To increase this ratio, the leakage current should be suppressed under HRS conditions. The leakage current is affected by the film phase, defect concentration, or energy barrier conditions [10,11,18,32]. More investigation is needed. Figure 5b displays the retention characteristics of the four resistance states. The three LRS and one HRS states exhibit excellent MLC behavior, maintaining their resistance values for up to 10^4^ s without any significant degradation.

To assess the capability of the Y_2_O_3_ RRAM as a synaptic component with long-term plasticity for the offline-learning-based neural networks, numerical simulations were conducted. Figure 5c illustrates the hardware systems, comprising three distinct neuron layers, constructed to recognize handwritten digit images provided by the Modified National Institute of Standards and Technology. The system involved 784, 512, and 10 neurons for the input, processing, and output signals, respectively. The three layers of neurons were interconnected through artificial synapses. Each single synapse cell in the network comprised five different RRAM devices, and the nonvolatile memory states of these devices were estimated based on the results shown in Figure 5c. During training, optimal distributions of synaptic weights were calculated through software simulation. These weights were then converted into feasible conductance values for the RRAM cell. Figure 5d shows the recognition accuracy of the constructed system after offline training for 60 epochs. The hardware neural networks based on the Y_2_O_3_ RRAM devices effectively classified the digit images with a high accuracy of approximately 88%. Although the recognition accuracy of the hardware system was slightly lower than that of the ideal software (about 92%), the recognition accuracy of the system can be simply enhanced by employing a continuous synapse cell comprised of more than five RRAM devices. This demonstrates that the proposed Y_2_O_3_ RRAM devices can be utilized as a memory component to achieve practical neuromorphic systems.

## 4. Conclusions

Sol–gel-processed Y_2_O_3_ RRAM devices were successfully fabricated on ITO/glass substrates. These devices exhibit conventional bipolar RRAM characteristics, eliminating the need for a high-voltage forming process. By increasing the set current compliance values, the resistance values obtained gradually decrease, allowing for the realization of intermediate resistance values within the LRS and HRS, rendering them suitable for MLC switching. By regulating the set current compliance values, pure Ag filament conductive filament formation was restricted. The dominant oxygen ion diffusion and migration within Y_2_O_3_ leads to the formation of O_V_ and Ag metal–mixed CFs between the two electrodes. The filament composition changes from pure Ag metal to Ag metal with O_V_, which is critical for realizing the MLC switching. The fabricated devices can effectively operate as an MLC with a capacity of two bits in one cell, utilizing three LRS and one common HRS. When applying a low compliance current to the device, the formation of CF is suppressed, leading to a narrower CF and a slight increase in the resistance of the switching layer. To evaluate the potential of the Y_2_O_3_ RRAM devices for neural networks, numerical simulations were conducted. The results demonstrate that hardware neural networks based on the Y_2_O_3_ RRAM devices effectively classify digit images with a high accuracy of about 88%. This indicates that the proposed Y_2_O_3_ RRAM devices can be utilized as a memory component to achieve practical neuromorphic systems.

## Data Availability

The data are available in a publicly accessible repository.
